# Education Research: Bedside Rounding Alliance for Internal Medicine & Neurology (BRAINs): A Trainee-Led Program to Promote Residents' Confidence With Neurology

**DOI:** 10.1212/NE9.0000000000200209

**Published:** 2025-03-19

**Authors:** Galina Gheihman, Prashanth Rajarajan, Marinos G. Sotiropoulos, Sarah Esther Conway

**Affiliations:** 1 Department of Neurology, Mass General Brigham, Boston, MA;; 2 Harvard Medical School, Boston, MA; and; 3 Department of Neurology, Brigham & Women's Hospital, Boston, MA.

## Abstract

**Introduction and Problem Statement:**

Patients with neurologic symptoms are often first assessed by general medical providers, many of whom endorse low confidence and knowledge in evaluating neurologic patients. To address this gap, we designed, implemented, and evaluated the Bedside Rounding Alliance for Internal Medicine & Neurology Residents (BRAINs) Program.

**Objectives:**

Program objectives for internal medicine residents (IMRs) were to develop an approach to obtaining a focused neurologic history and to performing a focused physical examination. Objectives for neurology instructors were to demonstrate a focused neurologic history and examination and practice observing learners examining patients and providing specific feedback.

**Methods and Curriculum Description:**

BRAINs paired neurology trainees (residents and fellows) with teams of 4–5 IMRs and subinterns for a 45-minute structured bedside teaching session bimonthly. Neurology instructors demonstrated a focused history and examination at the bedside for a patient admitted with a neurologic concern. IMRs then practiced a hands-on examination with real-time feedback. We evaluated the BRAINs program using a mixed quantitative-qualitative postsession survey. Neurology educators were surveyed on their teaching experiences.

**Results and Assessment Data:**

Seventy-four IMRs participated and completed the survey since 2022. Participants agreed that BRAINs met the learning objectives of developing an approach to obtaining a neurologic history (69/74, 84%) and performing a neurologic examination (71/74, 96%). IMRs felt more confident in taking a focused history (62/74, 85%) and performing an examination (69/74, 93%) after the session. Ninety-five percent felt that bedside teaching was more effective than traditional didactics. Eighteen neurology educators completed the survey. One hundred percent reported that BRAINs was an effective way to practice near-peer teaching, and 89% reported that it was more effective than traditional didactics. Feedback from IMRs and educators helped iteratively improve the initiative.

**Discussion and Lessons Learned:**

The BRAINs program offered a scalable and adaptable structured teaching session that accommodates learners at various levels and promotes near-peer teaching in the clinical setting. The program is not resource intensive and can be scaled to different specialties, populations of learners, and hospitals. The BRAINs program is an educational innovation that supports cross-departmental camaraderie, empowers near-peer educators, and equips IMRs with increased confidence in neurologic knowledge and skills.

## Introduction and Problem Statement

Patients with neurologic symptoms and concerns are often first assessed by general medical providers in the primary care, urgent care, or hospital setting. While the number of neurologists is slowly growing to meet the rising demand for access for neurologic patients, most diagnosis and management of uncomplicated neurologic diseases continue to be practiced by primary care physicians and medical hospitalists.^1^ Therefore, internal medicine residents (IMRs) who may go on to practice in these positions require training in neurology to ensure that they are knowledgeable, competent, and confident in obtaining a neurologic history and performing a neurologic examination.

Internal medicine residencies require training in neurology, as mandated by the Accreditation Council for Graduate Medical Education.^2^ However, in 1 study of US medical students' and IMRs' perceptions of 8 medical subspecialties, respondents felt that they had the least knowledge of neurology and that it was the most difficult medical specialty.^3^ A term for this phenomenon has been coined: “neurophobia,” a discomfort with assessing and treating neurologic concerns^4^—and it is common among not only medical students and residents but also primary care physicians.^5^

In 2021, we surveyed categorical IMRs at our institution about their experience with diagnosing and treating common neurologic conditions.^6^ Sixty-two percent lacked confidence in diagnosing and treating neurologic disease; half (52%) reported confidence with the neurologic examination. We also surveyed residents about how they preferred to learn neurology. IMRs preferred to learn through bedside teaching (89%), lectures (54%), online cases or modules (44%), and textbooks (10%). This represented an unmet need for developing infrastructure for IMR teaching in neurology.

In response, we designed and implemented the Bedside Rounding Alliance for Internal-Medicine & Neurology Residents (BRAINs) program. In this trainee-led program, neurology educators (residents and fellows) were paired with groups of IMRs to conduct a structured bedside teaching session twice monthly. Given that IMRs in our institution lacked confidence in performing the neurologic examination, we focused our initiative on improving their confidence and used the learning method (i.e., bedside teaching) that was most endorsed in the needs assessment. In this article, we describe this curriculum innovation and our initial evaluation of the program.

## Objectives

The objectives of the BRAINs program were as follows:

1. For medicine residents:
  - Develop an approach to obtaining a focused neurologic history and performing a focused neurologic physical examination for common neurologic symptoms
  - Practice common neurologic examination maneuvers and receive real-time feedback
2. For neurology residents and fellows:
  - Demonstrate an approach to obtaining a focused neurologic history and performing a physical examination for a specific patient case selected for rounds
  - Practice observing learners examine patients and provide specific feedback

The aims of the program evaluation were as follows:

1. Assess IMRs' satisfaction with the BRAINs program and self-rated confidence in the neurologic history and examination
2. Assess neurology residents' and fellows' satisfaction with the BRAINs program and self-rated confidence in teaching the neurologic history and examination
3. Understand ways to improve the BRAINs program to meet residents' needs

## Methods and Curriculum Description

### Study Population

The study included all IMRs of all postgraduate years (PGYs) (including preliminary and categorical interns) and subinterns (fourth-year medical students) who rotated through the general medical inpatient service at Brigham & Women's Hospital (BWH) from April 2022 to June 2024. Given that subinterns on their advanced medicine rotation at HMS function similarly to interns (regarding patient volumes and resident supervision), we felt that their experiences were similar to PGY-1 IMRs and included them in this study. For ease of readability, we include these subinterns in our results when referencing IMRs throughout the article. Neurology educators included neurology senior residents (PGY3 and PGY4) and fellows (PGY5 and beyond) who volunteered for the program. All neurology senior residents and fellows at BWH were eligible to volunteer to teach. Exclusion criteria were IMRs rotating on the general medical inpatient service who did not participate in the session or neurology senior residents and fellows who did not volunteer.

### Program Design

#### Session Timing

We worked with colleagues in the Department of Medicine to identify the best time and rotation to conduct the BRAINs program and selected a dedicated teaching hour on Thursdays from 4 to 5 pm for IMRs on the general medical inpatient service. We placed an open call for neurology trainees to volunteer as educators; trainees signed up for 1 or more sessions using an online shared Google Spreadsheet. One week before the session, the BRAINs coordinator (a senior resident running the program) emailed the neurology educator and the IMRs rotating on the general medical inpatient service about the program and reminded them of the program objectives and connected the neurology educator to the medical team.

#### Patient Selection

The neurology educator identified an appropriate patient for the session and spoke with the patient in advance to explain the initiative and obtain verbal consent to participate. Usually, a patient on the neurology inpatient ward service was selected. The educator reminded the patient that this experience was strictly for educational purposes. Patients had the right to decline participation at any time. The educator then emailed the team where to meet to conduct the teaching session.

#### Bedside Teaching

Most of the session was spent at the bedside; educators often started with a brief 1–2 line summary of the patient although the diagnosis, management, and/or imaging were not the focus. At the bedside, neurology instructors demonstrated a focused history and examination for a patient admitted with a neurologic concern. The patient case was used to select a targeted subset of the neurologic examination. For example, the educator might focus on assessing weakness, the cranial nerves, reflexes, or cognition (e.g., neglect), depending on the presenting symptom. Rarely, if a patient did not have neurologic examination findings, the educator used the session to demonstrate a basic screening neurologic examination and proper technique (e.g., review of confrontational strength testing and reflex testing). IMRs then practiced a hands-on examination with the patient and received real-time feedback and correction of technique from the neurology educator. Feedback was verbal and focused on proper examination technique or interpretation of examination findings. Neurology educators might correct positioning in performing the examination or comment on the sequence of testing (e.g., proper distance for visual field testing, options for facial strength testing, and testing fatiguability). All feedback was formative and shared in the spirit of improving the confidence of IMRs in their examination skills. Educators did not follow a specific feedback structure but were prompted in advance to demonstrate a focused examination and offer feedback on related maneuvers. After the practice, there was an opportunity to ask questions. Each session lasted 45 minutes.

### Evaluation

We evaluated the BRAINs program with a mixed quantitative-qualitative postsession survey. Surveys were administered through a RedCap link delivered by email. Five minutes at the end of the session were reserved for residents to complete the survey. Data were anonymous, and participation was voluntary and confidential. IMRs and neurology educators were given a $5.00 incentive gift card to the hospital cafeteria for completing the survey.

IMR participants rated to what extent the session achieved its objectives and their confidence with the neurologic history and examination. Neurology educators evaluated the quality and efficacy of the session regarding developing their teaching skills. Ratings were on a 5-point Likert scale (1: strongly disagree to 5: strongly agree).

### Data Analysis

Excel was used to compile quantitative data and report descriptive statistics. We collapsed ratings of strongly agree/agree into a single category of agree and ratings of disagree/strongly disagree into a single category of disagree. For open-ended responses, we reviewed resident feedback to understand what contributed to the effectiveness of the program and suggestions for future improvement. Descriptive statistics were reported as mean ± SD.

### Standard Protocol Approvals, Registrations, and Participant Consents

The BWH Institutional Review Board reviewed this study proposal and waived it from full review. Informed consent was presumed if residents chose to participate in the voluntary research survey. Residents could withdraw participation in the research component at any time, and participating in the teaching session did not require their participation in the research component.

### Data Availability

Data not provided in the article because of space limitations may be shared (anonymized) at the request of any qualified investigator for purposes of replicating procedures and results.

## Results and Assessment Data

### Participant Demographics

Seventy-four IMRs and 18 neurology educators participated in the BRAINs session and completed the survey over the research period. Approximately 3–5 IMRs participated at a time (range 2 to over 10). Fifty-four IMRs (73%) were able to attend the whole session (some IMRs were pulled away for part of the time for clinical duties). We were unable to report a response rate for the survey because we did not track overall attendance at each session. Some educators participated in multiple sessions. Of the 11 educators who indicated the number of BRAINs sessions they taught in, 5 of 11 (45%) taught 1 and 6 of 11 (55%) taught 2. Additional demographic data are provided in Table 1.

**Table 1:** Demographics of the IMRs and Educators Completing the Survey

IMR year	Number (%)	Educator year	Number (%)
Subintern	6 (8)	—	—
PGY-1	46 (62)	—	—
PGY-2	10 (14)	—	—
PGY-3	11 (15)	PGY-3	8 (44)
PGY-4	1 (1)	PGY-4	5 (28)
PGY-5 and up	0	PGY-5 and up	5 (28)
Total	74	Total	18

Abbreviations: IMR = internal medicine resident; PGY = postgraduate year

### Session Logistics

Educators reported teaching for an average of 41 minutes (41.5 ± 14.8). Among 18 topics reported, the primary neurologic concern/symptom or examination focus included the following in descending order (counts): hemiplegia (4), aphasia (2), dizziness and sensory change/brainstem stroke (2), leg weakness (1), diplopia (1), internuclear ophthalmoparesis (1), amyotrophic lateral sclerosis (1), functional/psychogenic nonepileptic seizures (1), transverse myelitis (1), vision loss (1), and subarachnoid hemorrhage (1).

### IMR Participant Experiences

**Figure 1:**
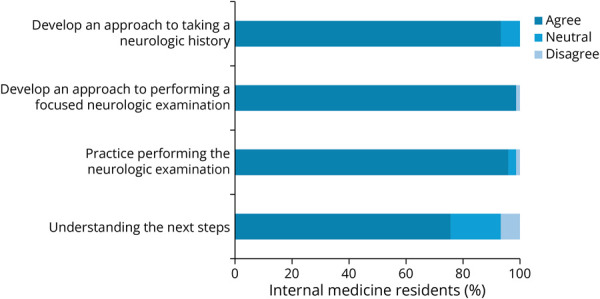
BRAINs Sessions Met the Program's Objectives, as Rated by IMRs Bar graphs show the percentage among IMRs who completed the survey who felt that the BRAINs sessions met the program's objectives. Percentages are shown on the x-axis. Agree, neutral, and disagree are shown in different shades of blue. BRAINs = Bedside Rounding Alliance for Internal Medicine & Neurology Residents; IMR = internal medicine resident.

**Figure 2:**
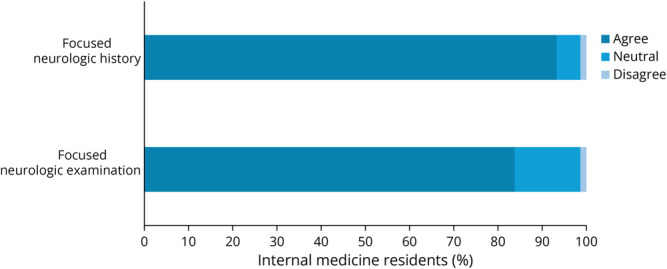
IMRs' Self-Rated Increase in Confidence With Obtaining a Focused Neurologic History and Completing a Focused Neurologic Examination After the BRAINs Session Bar graphs show the percentages among IMRs who completed the survey who felt that the BRAINs sessions increased their confidence in obtaining a focused neurologic history and completing a focused neurologic examination. Percentages are shown on the x-axis. Agree, neutral, and disagree are shown in different shades of blue. BRAINs = Bedside Rounding Alliance for Internal Medicine & Neurology Residents; IMR = internal medicine resident.

Most IMRs agreed that the BRAINs sessions met the program's objectives (Figure 1). The sessions also improved IMRs self-rated confidence in taking a focused neurologic history and performing a focused neurologic examination after the session (Figure 2).

One hundred percent (74/74) of IMRs rated the material covered as appropriate for their level of training, and 91% (67/74) considered the session length (45 minutes) to be appropriate. Ninety-eight percent (73/74) had a favorable impression of the program.

Ninety-five percent (70/74) of IMRs felt that the teaching session was more effective than traditional didactics, and 89% (65/74) found that it gave them an opportunity to get to know their neurology residency colleagues. Ninety-seven percent (72/74) of IMRs would recommend the BRAINs program to others. IMRs provided qualitative comments on the program. Thematic analysis revealed that IMRs appreciated the interactive nature of the session, learning from real patients at the bedside, being taught by a fellow resident, focusing on just 1 or 2 examination areas, and learning in a fun, nonthreatening environment. Sample quotations from residents are provided in Table 2.

**Table 2:** Qualitative Feedback From IMRs and Neurology Educators Highlights What They Appreciated About the Program

Qualitative feedback from IMRs (selected quotes)	Qualitative feedback from neurology educators (selected quotes)
**Interactive, bedside teaching** • I loved the bedside teaching and interactive nature of the session • Teaching at the bedside on physical exam maneuvers with real time feedback • Feedback in real time, great explanations • Hands-on practice with open Q&A • Great for bedside real time interaction with neuro colleagues and patients! Improved ... examinations a lot by getting hands on practice • I loved the hands on aspect and going systematically through ... motor exam **Near-peer teaching** • Learning from another resident, interacting with and discussing a real patient in person • I appreciated the ability to practice a neuro exam with a neuro resident. I appreciated the walkthrough of the localization and differential diagnosis **Focused teaching** • Ordering of case review/imaging and practicing skills, focused on one or two exam areas • Seeing how to approach a focused neuro exam and how that guides diagnostics • Focused session on a particular exam **Safe learning environment** • Correlating findings to imaging. Fun and non-threatening • Friendly neuro resident, comprehensive	**Natural bedside teaching** • It's really great using our own patients as examples for practicing different parts of the neuro exam • ... I had a patient with a specific neurological finding (INO). I asked the seniors on service for a patient and they identified this one. It allowed me to plan my teaching and structure it around this finding. **Near-peer teaching** • Demonstration and interaction with residents • The residents and medical students that were able to show up were engaged and interested in learning. Loved that atmosphere of engaged learners **Focused teaching and feedback** • Focused history and exam taking. Giving direct feedback on exam skills • I liked the informal nature and it was great to practice exam techniques and give feedback and tips • [Small enough groups that] everybody gets a chance to practice **Session length and organization** • The guidance ahead of time was excellent • Supportive organizers helped get the session set up and get it running! • The session length is just right—it's an appropriate amount of time to cover the session objectives

Abbreviation: IMR = internal medicine resident.

### Neurology Educator Experiences

Neurology educators were surveyed about their experiences. Eighty-nine percent (16/18) were able to demonstrate a focused history and examination for a specific patient, and 89% (16/18) were able to observe learners examining patients and provide specific feedback. Seventy-eight percent (14/18) felt that the length of the session was “just right.” Eighty-three percent (15/18) felt that the teaching experience was well organized.

One hundred percent of educators reported that the BRAINs program was an effective way to practice near-peer teaching skills, and 89% (16/18) reported that it was more effective than preparing and giving traditional didactic lectures. Eighty-three percent (15/18) stated that this teaching experience gave them an opportunity to get to know their medical residency colleagues better. All but 1 educator recommended this experience to others. Regarding areas for improvement, 39% (7/18) of educators agreed that they got enough support and preparation for the session while 58% (11/18) rated this statement neutrally.

Neurology educators provided qualitative feedback, including what worked well and what could be improved (Table 2). Like IMRs, neurology educators appreciated the interactive, informal nature of bedside teaching. They felt that they had sufficient guidance for leading the session and that session length was appropriate. Neurology educators appreciated engaging with colleagues and the opportunity to give feedback and develop as near-peer educators.

### Program Feedback and Iterative Change

Over 2 years of implementation, we learned important lessons. Iterative changes were made to the program in the early months of implementation based on IMR and educator feedback to improve participation in the program (Table 3).

**Table 3:** Summary of Changes Implemented for Program Improvement Based on Early Qualitative Feedback From IMRs and Neurology Educators

Program aspect	Change implemented
Session length and timing	Adjusted from 60 min from 4 to 5 pm, to 45 min from 4:15 to 5 pm, to allow for the IMR team to sign out from 4:00 to 4:15 pm. To save time, teams met the neurology educator directly at the bedside of the selected patient
IMR awareness of the program	Program mentioned during IMR orientation; email and calendar invite sent ahead of time to IMRs by the neurology educator
Patient selection	Initially responsibility of the medical team, then switched to the neurology educator to select a patient who is willing to participate
Topic focus	Introductory email sent ahead of time to the neurology educator, reminding them of program objectives (focusing on a portion of the examination rather than focusing on diagnostics, imaging, or management discussion)
Feedback survey	Five minutes were allocated at the end of the session for IMRs and neurology educators to fill out the postsession survey to increase survey participation (completed on phone by residents from link sent in reminder email)

Abbreviation: IMR = internal medicine resident.

#### Session Timing and Length

- The BRAINs session was offered during a dedicated afternoon teaching hour on the general medical inpatient service, integrating neurology exposure into the dedicated teaching time rather than searching for additional time in the curriculum.
- The session length was shortened from 60 to 45 minutes, allowing team changeover from 4 to 4:15 pm, with the session running from 4:15 to 5:00 pm.

#### IMR Awareness of the Program

- The session was advertised to the general medical inpatient teams as they began their rotation to raise resident awareness about the program. Reminder calendar invites were sent to both the IMRs and neurology educator to increase participation.

#### Patient Selection

- In an earlier iteration, we asked medical teams to select a patient on their service because we hoped that learning from an admitted patient on their team would be fruitful. However, teams were inconsistent with selecting a patient, and at times, no patients with neurologic symptoms were admitted on the general medical teams. Given this, the neurology educator selected a patient from the neurology service and consented the patient for participation. This allowed the educator to also prepare in advance by selecting a particular aspect of the neurologic examination to focus on.
- We found that teaching directly at the bedside was most useful. Rather than meeting in a conference room, teams met outside the patient room and began beside teaching after introductions, which allowed more time for the examination and real-time feedback.

#### Preparing and Supporting Neurology Educators

- To better prepare neurology educators for their role, we sent a template email summarizing program objectives and sample topics to help orient them to the program ahead of time. Educators were reminded to keep the teaching focused on 1 or 2 actionable skills (e.g., examination technique) related to the chief concern. While some IMRs had questions about imaging and management, this was not the major focus of our initiative.
- We expanded the teaching opportunity to neurology fellows to increase access.

#### Survey Administration

- To increase survey participation, 5 minutes were allocated for IMRs and neurology educators to fill out the postsession survey.

## Discussion and Lessons Learned

In our study of a bedside teaching initiative of neurology examination skills for IMRs, we found that BRAINs was an effective teaching program that leveraged near-peer education in the clinical setting with real patients and a supportive environment. IMRs preferred it to conventional lecture-based didactic sessions and left the session with overall increased confidence in their approach to common neurologic concerns. Neurology educators found it an excellent method to practice their near-peer teaching skills. The program did not require significant planning; was not resource intensive; and can be scaled and adapted to different services, learner populations, and hospitals.

The diagnosis and management of most uncomplicated neurologic diseases continue to be practiced by primary care physicians and medical hospitalists, rather than specialty neurologists.^1^ Moreover, there is a growing shortage of neurologists in the United States, with the gap expected to grow to 19% by 2025.^7^ As neurology providers, we have a duty to expand access to quality care to patients with neurologic concerns and diseases. One opportunity to do so may be in the training of front-line providers—primary care doctors, hospitalists, emergency room providers, etc.—to be more comfortable with and confident in their neurologic history taking and examination skills. The BRAINs program addressed this general need and adapted to the local needs of our specific setting and to the learning preferences of our target group of IMRs.

A strength of our intervention was that it was based on a targeted needs assessment of IMRs in our own institution, which revealed that 62% lacked confidence in diagnosing and treating neurologic disease and 48% lacked confidence with the neurologic examination.^6^ We designed the intervention following adult learning principles,^8^ including making the learning interactive, clinically based and clinically relevant, hands-on, and nonevaluative. We also surveyed residents about how they preferred to learn neurology. Using Kern's 6-Step Approach to Curriculum Development,^9^ we used the general and targeted needs assessment to select our educational structure, implementation plan, and assessment. The choice to run bedside teaching sessions, for example, was informed by this needs assessment. Feedback from the initial cycles of the program helped improve it over time. IMRs preferred the BRAINs program to traditional didactics and cited the interactive, hands-on nature among its strengths. Because the sessions were not evaluative, we hypothesize that this created a safe learning environment in which participants were able to practice and receive real-time, directly observed feedback. This is important because medical students^10,11^ and residents^12-16^ report a lack of feedback opportunities during clinical rotations.

Our study used near-peer teaching, a teaching strategy where the educator is also a trainee but is more advanced or has specialized knowledge compared with their student peers.^17,18^ Near-peer teaching is effective and efficient for teaching medical students^19^ and residents^20^ in the classroom and at the clinical bedside. In neurology education specifically, peer educators have been found to be effective teachers of basic neurosciences,^21^ neuroanatomy,^22^ acute neurology simulations,^23^ and on the clinical wards^24^; however, studies investigating teaching of the neurologic examination specifically are lacking.

Near-peer education benefits the learner. First, there is a similarity of age and training stage among near-peer educators and learners, which may support positive learner outcomes.^25^ Near-peers can understand the perspectives of their learners and may be more approachable,^26^ thus perceived to be less threatening than faculty educators. This may promote a more comfortable learning environment, reducing stress and cognitive load. This is particularly important in teaching neurology because many trainees suffer from “neurophobia,” a term coined by Jozefowicz^27^ to describe medical students' fear of neurology.^28^ Health care professionals and medical students often describe neurology as the most challenging subject in clinical medicine and feel unskilled in managing neurology patients.^3^ Students report insufficient clinical exposure and inadequate clinical teaching as principal drivers of neurophobia. Several strategies have been suggested to overcome neurophobia including incorporating active learning experiences,^29^ using engaging teaching methods such as problem-based or team-based learning, and incorporating near-peer resident teaching at various stages of neurologic education.^3^ Reassuringly, while peer educators may have slightly less knowledge than faculty, learning outcomes when taught by peers and faculty are nonetheless overall similar.^26,30-33^

Another advantage of near-peer teaching is that it benefits the resident-teacher. The trainee teacher is able to consolidate and reinforce their knowledge base^34,35^; practice communication skills, professionalism, and interprofessional teamwork^36^; develop their own learning; and advance their skills as an educator, which is an essential competency for all physicians.^37^ Experience in teaching is a requirement in graduate medical education^38^ and, increasingly, in undergraduate medical education.^39^ BRAINs offered an opportunity for residents to hone these skills. Neurology educators agreed that the program was an excellent method to practice near-peer teaching skills and felt that they received adequate preparation and support. A limitation of using near-peer educators is the variability in the preparation of and expertise of trainees, both regarding content knowledge and teaching ability. To better prepare educators, we sent a template email with information ahead of time, orienting them to program objectives to increase consistency among sessions and reduce variability. In this iteration of the program, we were not able to offer direct observation and feedback for our neurology educators. We hope to expand the program and offer near-peer observation and feedback by pairing neurology educators with a peer or faculty so that they may also undergo observation and receive feedback to improve over time.^40^

An important consideration for our initiative is its sustainability. The program did not require significant planning; was not resource intensive; and can be adapted to different services (e.g., emergency medicine and pediatrics), learner populations, and hospitals. The program used existing teachers (residents on the ward services) and learners in their place of work and was adaptable to working with admitted patients. The main resource required was time (although this can be challenging to protect on busy clinical services). The program was entirely trainee-led. We have sustained the program over 3 years in our institution by passing down program leadership among generations of senior residents. We have maintained collaboration with the Department of Medicine through a faculty liaison role and collaboration with a representative medicine chief resident in running the program, a role that has also been passed down through 3 generations of chiefs. While many training programs cite limited faculty^41-43^ time as a barrier to implementing educational interventions, we were able to circumvent this using trainee teachers. One educator was able to provide a small team-based learning experience, with enough time to offer hands-on practice and individualized feedback for all learners.^18,34,44^

Teaching at the bedside is losing its centrality in the clinical learning environment, as access to data and imaging grows and begins to monopolize patient assessment. Nonetheless, taking the teaching back to the bedside remains a key principle of effective physical examination teaching practice.^45^ Proponents of physical examination teaching emphasize the need for bedside clinical skills practice and development, including approaching patients with a hypothesis-driven examination.^46^ Breaking down the neurologic examination into components may likewise be helpful for reinforcing individual maneuvers and a hypothesis-testing approach.^47^ Our intervention prepared educators using best practices in bedside teaching, including starting with an introduction, setting a focused intention, offering coaching with corrective feedback, and summarizing what was learned.^48^

In addition to improving individual learners' understanding of neurology history taking and examination skills, our intervention has the potential to improve interdepartmental understanding and camaraderie. While previous work has shown that implementing rotations on neurology services may help clarify appropriateness of and expectations for neurology consults,^49^ this may not be feasible for all programs to implement. BRAINs offered a lower commitment opportunity that may still build non-neurology residents' confidence and competence and augment interdisciplinary communication. The program could be expanded to other specialties and learner groups that frequently interface with neurology (e.g., emergency residency physicians, primary care physicians, cardiologists, and advanced practice providers) and other topics overlapping with neurology (e.g., neuroinfectious disease skills, neurocardiology, and neurorheumatology).

Future areas of research include studying the longitudinal impact of the program on clinical practice and whether learning is sustained over time. We assessed residents' self-report of knowledge and confidence but did not evaluate changes in behavior. Of note, self-reported confidence and competence to perform a behavior are distinct. We were interested in whether this intervention could increase self-reported confidence because this was found to be low among IMRs in our targeted needs assessment.^6^ Ideally, follow-up studies would observe IM residents in practice to evaluate their use of appropriate examination maneuvers and assess competence objectively (Kirkpatrick level 3 outcome). It would also be important to determine whether our measure of increased self-reported confidence is correlated with improved competence and ultimately with patients' clinical outcomes. In future, we hope to assess whether the BRAINs program helped IM residents obtain accurate histories and perform accurate examinations to improve or change management (Kirkpatrick level 4 outcome). Future iterations could also include standardized competency examinations before and after the intervention or observational assessments of IMRs' clinical practice to further assess the program's impact. We also hypothesize that as IM residents gain confidence in their neurology skillset, this may affect consultation volume or the quality and/or appropriateness of consultation questions in the inpatient setting and/or referrals to neurology in the outpatient setting. Future work could examine this, as well as whether BRAINs improves interdisciplinary collaboration among the IM and neurology residents and our departments.

We also noted unexpected benefits of the program for our patient participants and some attending physicians on the IM side who participated. We did not study the effect on patients, but future qualitative research could explore their experiences as patient-educators in the BRAINs program. Although IM attendings were not the primary target of our intervention, some did participate with their teams, and anecdotal evidence suggests that they benefited from the refresher. It may be worth exploring a BRAINs-like intervention for attendings and expanding the study to include them and their perspectives. Often, neurology consultations may be driven by the attending physician,^50^ and/or attendings can model the neurologic examination for their team; hence, their confidence in this skillset may have an important role-modeling effect on IMRs. We also hope to introduce peer-to-peer observation for neurology educators and study the effect of formal feedback on their teaching in supporting their professional identity development as an educator. Our study describes the experience of a single academic medical center in an urban environment and may not be generalizable to other institutions. Our institution has a dedicated neurology ward service for inpatients admitted with neurologic concerns. For institutions without a dedicated ward, our approach may need to be adopted with patients on the neurology consult service, for example. We have a large neurology residency program and multiple fellowships, making neurology educators readily available. In centers without this possibility, neurology faculty could play the role of the educator. For nonacademic institutions without dedicated neurology services, the neurology consultant may offer a similar bedside rounding program for internal medicine hospitalists or advanced practice providers. We have an established liaison relationship with our Department of Medicine, given that their residents rotate with us; this existing interdepartmental camaraderie may have increased buy-in for our program. Our study was limited by the fact that we assessed residents' self-reported confidence in their history taking and examination skills after a single session; we do not know whether this self-perceived increased confidence translates into behavior change or whether it can be sustained over time. We report data from collected surveys only; there may be response bias because we do not know whether the experiences with the intervention differed among survey nonresponders. Finally, there was variability among patients, educators' teaching styles, and the group size and interest of the IMRs—this may undermine our ability to report on objective outcomes of the program. We recognize that more systematic preparation of neurology educators, including orientation to program objectives and use of a standardized feedback process, may help reduce variability between sessions. However, the robustness of the program and the perceived effectiveness and organization across these elements of variability support the feasibility of this intervention. It is adaptable and effective, despite changes in learners, patients, and educators.

We designed, implemented, and evaluated an innovative, adaptable, trainee-led bedside teaching initiative of neurology examination skills for IMRs that improved learners' self-rated confidence in neurologic history taking and examination skills. The BRAINs program offered a scalable and adaptable structured teaching session that accommodated learners at various levels and promoted near-peer teaching in the clinical setting, all in a safe learning environment. The program was not resource intensive; did not require significant advanced planning; and can be scaled to different specialties or services (e.g., emergency department and pediatrics), populations of learners, and hospitals. The BRAINs program supported cross-departmental camaraderie, empowered near-peer educators, and equipped IMRs—who are often the first to assess neurologic patients—with increased confidence in neurologic knowledge and skills.

## Glossary

BRAINs: Bedside Rounding Alliance for Internal-Medicine & Neurology Residents
BWH: Brigham & Women's Hospital
IMR: internal medicine resident
PGY: postgraduate year
